# Assessing the implementability of telehealth interventions for self-management support: a realist review

**DOI:** 10.1186/s13012-015-0238-9

**Published:** 2015-04-24

**Authors:** Ivaylo Vassilev, Alison Rowsell, Catherine Pope, Anne Kennedy, Alicia O’Cathain, Chris Salisbury, Anne Rogers

**Affiliations:** Faculty of Health Sciences, University of Southampton, Building 67, University Road, Highfield, Southampton SO17 1BJ UK; University of Southampton, Building 44, Highfield Campus, Highfield, Southampton SO17 1BJ UK; Health Services Research Section, Medical Care Research Unit, ScHARR, University of Sheffield, 30 Regent Street, Sheffield, S1 4DA UK; Office Room 1.01b, Canynge Hall, 39 Whatley Road, Bristol, BS8 2PS UK

**Keywords:** Telehealth, Self-management, Chronic illness, Intervention, Mechanisms, Implementation

## Abstract

**Background:**

There is a substantial and continually growing literature on the effectiveness and implementation of discrete telehealth interventions for health condition management. However, it is difficult to predict which technologies are likely to work and be used in practice. In this context, identifying the core mechanisms associated with successful telehealth implementation is relevant to consolidating the likely elements for ensuring *a priori* optimal design and deployment of telehealth interventions for supporting patients with long-term conditions (LTCs).

**Methods:**

We adopted a two-stage realist synthesis approach to identify the core mechanisms underpinning telehealth interventions. In the second stage of the review, we tested inductively and refined our understanding of the mechanisms. We reviewed qualitative papers focused on COPD, heart failure, diabetes, and behaviours and complications associated with these conditions. The review included 15 papers published 2009 to 2014.

**Results:**

Three concepts were identified, which suggested how telehealth worked to engage and support health-related work. Whether or not and how a telehealth intervention enables or limits the possibility for *relationships* with professionals and/or peers. Telehealth has the potential to reshape and extend existing relationships, acting as a partial substitute for the role of health professionals. The second concept is *fit*: successful telehealth interventions are those that can be well integrated into everyday life and health care routines and the need to be easy to use, compatible with patients’ existing environment, skills, and capacity, and that do not significantly disrupt patients’ lives and routines. The third concept is *visibility*: visualisation of symptoms and feedback has the capacity to improve knowledge, motivation, and a sense of empowerment; engage network members; and reinforce positive behaviour change, prompts for action and surveillance.

**Conclusions:**

Upfront consideration should be given to the mechanisms that are most likely to ensure the successful development and implementation of telehealth interventions. These include considerations about whether and how the telehealth intervention enables or limits the possibility for relationships with professionals and peers, how it fits with existing environment and capacities to self-manage, and visibility-enabling-enhanced awareness to self and others.

## Background

The ubiquitous growth in technologies and devices for illness management has been accompanied by numerous studies evaluating the effectiveness and implementation of telehealth interventions. Despite this growing body of evidence, it is difficult to predict which technologies are likely to work and will be used in practice. Existing evidence points to the need for technologies to align closely with wider networks that facilitate collective efforts to enhance individual self-management [1]. However, there is a still a need to identify the mechanisms underlying the normalisation of technologies in the life worlds of patients [2-4]. The objective of the evidence synthesis presented here was to review and integrate evidence about telehealth interventions, including the provision of support and care at home and monitoring patient status at a distance using audio, video, web-based, and other technologies [5]. The focus was on identifying the structuring factors likely to promote implementation that should be taken into consideration when developing and deploying telehealth interventions.

Telehealth has been found to have a positive impact on social support [6,7], compliance [8], education [7,9,10], behaviour change and better self-management [9,11,12], and reduced burden on the individual and services [13,14]. The latter is particularly the case where interventions are telephone, computer, or internet-based [15,16] because they are simple to use and familiar [13,17]. Overall, patients appear more positive than professionals about using telehealth [17,18]. In a related field, Ziebland and Wyke [19] identified a number of domains through which online patients’ experiences could affect health (finding information, feeling supported, maintaining relationships with others, affecting behaviour, experiencing health services, and visualising disease). The gaps identified by previous reviews confirm the need for more in-depth understanding of how telehealth interventions mediate to improve health and become embedded in people’s everyday lives [18]. While many evaluation studies focus on the technology itself or the individual recipient in explaining (un) successful implementation in a field where technologies are being developed and introduced at a rapid pace [20], there is often little generic guidance about the likely impact in terms of acceptability and appropriateness that can be considered by those at the point of devising new technologies. While it is relevant to understand telehealth interventions in context, retrospectively analysing their contribution to patient care, it is useful to find ways to identify core features that can assist with telehealth intervention design and deployment. Here, we are interested in exploring interventions that could be delivered as part of self-care support for long-term (chronic) health conditions at the interface between populations and primary care. Our aim was to move beyond more quantitative, trial-based evidence (the primary resource that currently informs development) to include qualitative evidence in thinking about telehealth. Drawing together research conducted as part of two separate but related projects, the Healthlines Study (http://www.bristol.ac.uk/healthlines/) and the EU Wise programme [21], we posed the question: how (through what processes and mechanisms) can telehealth improve the health and well-being of people with long-term conditions (LTCs)? This paper addresses implementation of evidence-based practice of telehealth which crosses an interface between formal clinical settings and peoples’ everyday lives. The paper brings together two studies and aims to identify and then test the core mechanisms underpinning telehealth interventions by understanding how telehealth can improve the health and well-being of people with a range of LTCs.

### Theory development and theory testing

Theory development and theory testing were conducted in two stages. In stage 1, we used a realist synthesis approach to identify characteristics of telehealth interventions that had a positive impact on the facilitation of health and well-being of people with LTCs. The team of researchers (AR, CP, and AC) revisited the three reviews on telehealth interventions for people with LTCs undertaken for the Healthlines Study. These included a) meta-review of 16 systematic reviews centering on the topic of LTCs, published between 2005 and 2010; b) a meta-review of 20 reviews on depression, published between 2005 and 2010; and c) a meta-synthesis of 29 qualitative studies of interventions for LTCs, published between 2000 and 2010. These three reviews were conducted to provide the evidence base for a telehealth intervention targeting people with long-term conditions [17]. Our searches were of Medline, Embase/AMED, PsycInfo, Web of Science, DARE, and the Cochrane Library. The first review was designed to identify existing, broad, evidence about interventions for LTCs. The second two reviews were conducted to plug gaps in knowledge, specifically about depression (one of the target disease groups for the Healthlines intervention) and to capture the qualitative evidence which had not hitherto been synthesised. The interventions included telephone-based, telemonitoring and computerized, and web-based forms of telehealth. These included real-time (synchronous) and asynchronous (that is, email) interventions offered with and without healthcare professional input. Further methodological detail necessary to reproduce these reviews is provided in a report [17] and a working paper from the study [18] available here http://www.bristol.ac.uk/healthlines/. This literature provided an overview of the evidence for telehealth interventions aimed at adult patients in home settings. Overall, the reviews provided information about outcomes ranging from specific changes in clinical features (for example, reduced hypertension) and treatment compliance as well as on quality of life. Some also provided information on financial savings and many discussed acceptability and satisfaction with interventions. These outcomes demarcate the success or otherwise of an intervention—in effect they tell us if it ‘worked’. While these earlier reviews improved our knowledge about which interventions worked and which did not, they could not address the question *why* and through what mechanisms they worked. Thus, subsequently, a realist synthesis approach was chosen because of its suitability for conceptual development and theory building. In analysing the literature, we followed Pawson’s seven stage model: identify the question and clarify the purpose of the review, theory elicitation, search the evidence, appraisal, extract the results, synthesise findings, and draw conclusions and make recommendations. Realist synthesis was an iterative rather than a linear process where we compared findings from different studies, looking for examples which challenged, refined, or supported the theories identified [22,23].

We re-read the review literature to inductively identify potential mechanisms associated with successful interventions. We discussed these amongst our team, re-examining the literature for confirmatory and dis-confirmatory evidence and refining our ideas in a process that resembled qualitative thematic analysis. Three explanations or theories, which suggested how telehealth worked to improve health, were identified from this analytical process. These emerged from developing propositions about what *might* be important to the success of interventions in the context of the evidence we had read and synthesised. Our emergent theories about the core mechanisms underpinning successful telehealth interventions from this review work were as follows:

1. *Relationship. Relationships or connections between people (patients, peer groups, and/or lay and professional carers) are a necessary component of telehealth interventions*.
2. *Fit. The extent to which a telehealth intervention can be integrated within everyday life and health care routines determines the success of deployment/adoption*.
3. *Visibility*. S*ystems which increase the visibility of symptoms or health problems to self or others impact positively or negatively on the adoption of telehealth interventions depending on, for example, whether patients might want anonymity or not.*

The three mechanisms that we describe are relevant for assessing the likely implementability of existing interventions and for developing new ones that are more likely to be successfully implemented. These can be used as a basis for developing a set of sensitising concepts when consideration is being given to introducing new telehealth interventions. Stage 2 provided an opportunity to test our propositions against additional and more recent qualitative studies. We cast the net wide in stage 2 to ensure that we could include a range of different technologies, deliberately including newer innovations (for example, apps) that had not been examined in the earlier literature and populations that would help confirm or disprove our conceptual hypotheses about the mechanisms that underpinned successful telehealth interventions. If the mechanisms could be shown to stand up to this testing, then we could have more confidence in their application to any new telehealth interventions. In this paper, we report the theory testing results.

## Methods

For this review, we searched papers published between 2009 and 2014 which focused on COPD, heart failure, and diabetes. These three conditions were chosen as exemplars of LTCs that had high incidence and growing prevalence, which often co-exist with other conditions and to which there are increasing aspirations to manage through telecare interventions [24]. Papers for review were identified from searches in PubMed and the Web of Science. We looked for qualitative papers focusing on telehealth, telecare, self-management, diabetes, heart failure, COPD, and related conditions and risk factors. Papers were included if they reported studies conducted in OECD countries. While most of the papers selected for this review described successful interventions, this was not one of our selection criteria. This was because our interest was in understanding the processes that contributed to successful interventions, and therefore both successes and failures could potentially be equally revealing of underlying processes.

**Figure 1:**
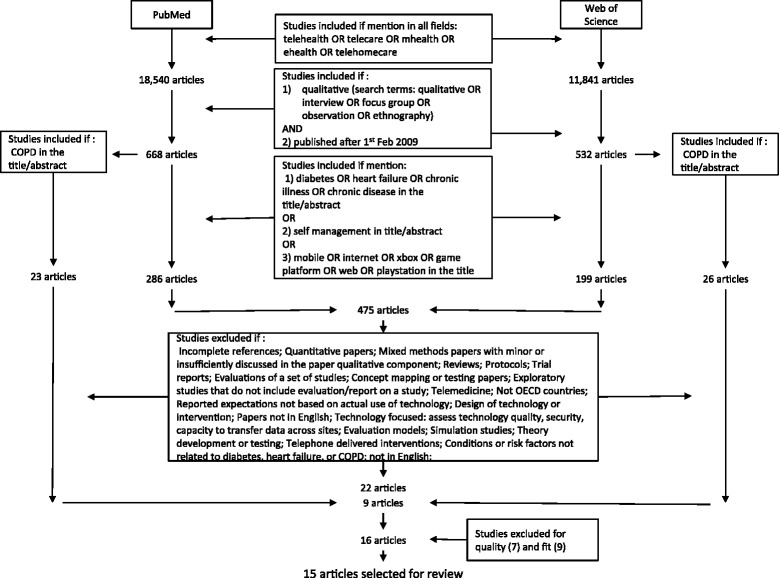
Review papers selection process. COPD, chronic obstructive pulmonary disease; OECD, Organisation for Economic Co-operation and Development.

Three reviewers (AER, AK, and IV) independently reviewed abstracts to agree on papers for full-text retrieval. Where there was doubt about a paper, the full-text paper was retrieved. Thirty one papers were reviewed for quality and fit using the CASP criteria [25] and after exclusions, 15 papers were included (see Figure 1). We designed a data extraction form that included a) background information about each paper (study setting, rationale, aims, research questions, sampling, data collection, and analysis), and b) key findings and themes identified by the authors. Each paper was analysed using the three mechanisms identified in stage 1, after which the papers were systematically compared (see Table 1). All three reviewers reviewed the full papers. The findings that were independently arrived at were then discussed and refined in an iterative process. The findings and their interpretation were then discussed within the whole team of researchers (AER, AK, IV, CP, AR, and AC).

**Table 1:** **Data extraction form for reviewed papers**

**Title and authors**	**Sample, setting, and aims**	**Key findings**	**Effective**	**Relationships**	**Fit**	**Visibility**
1. Improving diabetes care for young people with type 1 diabetes through visual learning on mobile phones: mixed-methods study; Froisland DH, Arsand E, Skarderud F.	Twelve participants (seven girls and five boys) aged 13 to 19 years; Norway; 1) To explore how applications for mobile phones can be used in follow-up of adolescents with type 1 diabetes, and (2) to use the findings to guide further development of the applications and as a basis for future studies	Pictures and photos of food taken by and stored on mobile phones important in increasing understanding of link between food, insulin, and blood sugar. SMS access to healthcare professionals a safety net and allowed easy access Visualisation access software changes	Yes	Access: The healthcare professionals (HCP) through texts—positive instrument for bi-directional contact with healthcare providers ‘I liked the project and the follow-up. I could send an SMS whenever I wanted. I got an answer within half an hour. I especially liked the SMS—in the Netherlands, where I lived prior to this, I knew I could call, but I like the message system a lot better*.*’ Improves relationships as this offers more control over timing of access; close substitutes to face-to-face encounters not necessarily desirable; here, SMS is preferred to a telephone call; more control over managing relationships	Mobile phone use nearly ubiquitous in adolescents, therefore, fits with daily life and skills in this generation Some problems with the software and need for improvement. Battery use problem ‘I didn’t like the diagram thing [in the Diamob apps]. It was a mess, and I didn’t like that it could not be tailored to each patient…And for instance I think it is stupid that it [the pictograms for activity] only marks sitting, lying, standing, and training, but maybe you don’t do the physical activity, and then, after an hour, it is time to go for a run.’	Visualisation: Pictures based diary of food better than SMS words and lots of information. Provided visual feedback of how what was eaten linked to insulin dose and blood sugar—led to improved understanding ‘It is just to browse back in the picture diary and look at how much [insulin] I actually needed to the food I had eaten, that is an advantage…’
2. Opportunities and challenges for smartphone applications in supporting health behaviour change: qualitative study; Dennison L, Morrison L, Conway G, Yardley L.	Young adults: 19 students and staff at a university, focus groups; UK; a series of focus groups with healthy young adult smartphone users to explore: (1) their existing experiences of using health-related smartphone apps, and (2) their views about a range of different features, technologies, and capabilities that characterize currently available or future apps. We sought their views on features that might support them in making changes to behaviours relevant to health, factors that contribute to interest in apps, willingness to use the apps, and issues leading to disinclination to using the apps	Apathy, concerns, and frustrations around health apps Checklist of valuable features Challenges for acceptable and effective behaviour change apps	Yes	Behaviour change, apps, and social networks Decisions and actions related to healthy lifestyle a private activity—socially undesirable ‘If this popped up, I think people would laugh at me’ Not to be used by linking to social network sites in a general way, ok if with specific group working to same goals. Credibility and accuracy Apps developed by experts preferable	Smartphones as valuable information sources Already used regularly for health information. Used ‘on the go’ Scepticism over context sensing Technology to detect location, mood, social situation, and activity levels to offer advice—not wanted—too gimmicky and intrusive and inaccurate ‘If it gets it wrong, you would automatically get really irritated by it…I think the risk if getting it wrong would be really annoying and I’d probably delete the app Necessity of efficiency and convenience Need to be well integrated with how phones used naturally—not using up too much space or power ‘Its quite easy to lose interest really because it is quite an effort and nobody wants to spend all their life writing down what they want on their phone Disposability of apps	Tracking progress and raising awareness ‘most people have their phones with them most of the time, so if you’re out and about and want to check how much you’re doing or what you’re burning when you’re walking, it’s a good idea’ Can use to set targets Records of tracked behaviour data could prompt healthier behaviour. But disappointing results could be negative ‘You’d probably be like, “oh well I might as well give up then, it’s too depressing’ Visibility not only about relations to others (anonymity, surveillance) but also relation to self (enhanced awareness and knowledge does not necessarily lead to better control of symptoms here). Useful prompts or harassment? ‘I would really like it, I think I need pushing into doing anything’ Nagging or harassing could lead to deleting app Motivation and Necessity of behaviour change Person needs to be already committed and signed up to it. Privacy and security concerns Worry about data getting into hands of third party. Some phone capabilities creepy and intrusive Keeping control over apps
3. Gender differences in diabetes self-management: a mixed-methods analysis of a mobile health intervention for inner-city Latino patients; Burner E, Menchine M, Taylor E, Arora S	Eight people, 75% identified as Latino; USA; Focus groups to examine how texts impacted diabetes self-management	Gender impacted the patients’ perceptions of the program, the challenges they faced and their individual management strategies.	No	Dietary self-efficacy differed by gender Men lacked knowledge and skills about diet. Women thought men needed more help from text-MED Women more reliant on info from social sources ‘Trusted friends and family. I have a really good friend; she is actually a nurse, so she’s the one that gives me a lot of advice. She has diabetes, too.’ Presence of peer support relevant, but might need to be tailored; different needs related to gender here, so presence of generic peer support might not be sufficient	Health information sources differed by gender “I like to read; I don’t really talk to anyone about that.” Desired content of educational materials differed by gender ‘A topic, I can’t speak for ladies, but for males, um, health symptoms as far as performance.’	
4. Usability evaluation of an online, tailored self-management intervention for chronic obstructive pulmonary disease patients incorporating behaviour change techniques; Viola Voncken-Brewster, Albine Moser, Trudy van der Weijden, Zsolt Nagykaldi, Hein de Vries, Huibert Tange	Eight COPD patients; Holland; Evaluate and improve usability of the eHealth intervention	Need for improvements in layout, navigation, and mostly in content	To some extent; suggestions for improvement		Content Length of program—too long Feedback not appropriate and needed to be tailored to severity of condition ‘If this is for people like me, there should be adjustments for functional limitations. Here they talk mainly about the possibilities, about people who are mobile etc., but the people who cannot get out of the house, for those adjustments should be made.’	Behaviour change module awkward for some ‘I find that hurtful… everybody has their own motives, you should not talk to people about that. I almost find that patronizing.’
5. Continuity, but at what cost? The impact of telemonitoring COPD on continuities of care: a qualitative study; Fairbrother Peter, Pinnock Hilary, Hanley Janet, McCloughan Lucy, Sheikh Aziz, Pagliari Claudia, McKinstry Brian	Thirty-eight COPD; Scotland; qualitative study nested in RCT to explore views of patients and healthcare professionals on telemonitoring—focus on impact on continuity of care	Relationship-based continuity of care important in delivery of telemonitoring services Operational challenges of ‘bolting on’ to existing usual care	Yes Continuity identified as a challenge	Reassurance, accessibility, and trust ‘Someone watching over them I think it's very good. It makes you feel like somebody’s looking after you. If anything goes wrong, you can get in touch with them any time you want … you’ve got the confidence that they're going to get something done. I can't fault them anyway.’ Frequency of contact and approachability of telemonitoring professional led to bonds of trust. Intermediary between patient and GP—bridged barriers ‘I’d say you get better [service] because if [telemonitoring professional’s first name] comes on the phone and she’ll say “I think you're needing to speak to the doctor”, she’s just giving me a warning that she’s going to get the doctor to phone me. And they’ll either say “Well, I think you’re needing to have some antibiotics” or “I think maybe we should pop over and just see you.”’ Practitioners felt they got to know patient better Technology not a substitute for trust based face-to-face contacts here but builds trust where there was no personal relationship before its introduction (the presumption that technology is a threat to existing relationships is not necessarily correct) Discontinuities Little information sharing in professional teams Issues of cost and continuity Problems when service centralised—leads to poor service ‘You’re on the phone [to NHS24] for about an hour. Really, it’s a joke … I mean at weekends usually if he’s taken ill he tried to hang off to the Monday.’	Records management and data interoperability Lack of interoperability between telemonitoring system and existing patient information. Increased volume of paperwork for telemonitored patients	
6. Exploring telemonitoring and self-management by patients with chronic obstructive pulmonary disease: a qualitative study embedded in a randomized controlled trial; Peter Fairbrother, Hilary Pinnock, Janet Hanley, Lucy McCloughan, Aziz Sheikh, Claudia Pagliari, Brian McKinstry	Thirty-eight COPD; UK; to explore patient and professional views on self-management in the context of telemonitoring in chronic obstructive pulmonary disease	Compliance medical model of self management paradoxically promotes dependence on professionals, patients considered that telemonitoring empowered self management by enhancing understanding of COPD	Yes; But not as envisaged	Telemonitoring data used to validate contact with healthcare professionals. Increased accessibility to validating knowledge overcame reticence to contacting professionals placing the patient in a more equal relationship to negotiate contact and access to resources (exactly the same as Rogers et al. 2011) Relationship between professional and patient remains one of compliance	Easy use of O2 saturation measurements to inform decisions about capacity to undertake domestic activities such as household chores family excursion. Reinforced attempts to access services ‘. . .you still get the same attention [as “usual care”]. In fact, I’d say you get better because if [telemonitoring staff member] comes on the phone and she’ll say “I think you’re needing to speak to the doctor”, they’ll phone me right away—within 15 min of her phoning… And they’ll either say “well, I think you’re needing to have some antibiotics” or “I think maybe we should pop over and just see you and see what you’re like”.’ (Female, 69 years old) Many found it helpful to know their oxygen saturation and to learn their ‘normal’ range by identifying telemonitoring data trends over time. ‘I’m okay from 87% (oxygen saturation) upwards and I never get better than 92. Even when I’m very well, I never get better than 92. But I go out and about and I do what I need to do and I manage it by walking…’	A means of determining state of health that was empowering. ‘It gives me a lot more independence. I am not dependent on making the judgment myself. You’re using measurements which normally wouldn’t be available to me as a patient. . . that’s good.’ Validation of self medication decisions
7. Diabetes connected health: a pilot study of a patient- and provider-shared glucose monitoring web application; Alice J. Watson, Joseph C. Kvedar, Basmah Rahman, Alexandra C. Pelletier, Gregory Salber and Richard W. Grant	USA; Real-time sharing of blood glucose results with providers could improve communication and lead to more timely medication titration. New technology platforms are available to support the delivery of innovative models of care delivery	Patients satisfied and its acceptable	Yes; Primarily in terms of acceptability	The DCH application facilitated greater communication between patient and provider. ‘The system helped facilitate communication. It can help get rid of appointments to the doctor, and medication adjustments can be more immediate.’ ‘Program was good for providing feedback to provider.’ ‘My provider discussed the journal page during my visit	‘Found it kind of helpful and fun to track what I was eating and how that affected my readings … in particular when I was supposed to have a medication change’ The use of the product increased patients’ general awareness of their blood sugar and its changes. ‘The ability to notice trends on the days of the week and times of the month.’ ‘Graphs are a quick way of seeing how [blood sugar] fluctuates.’ ‘I found it much easier to keep track of both my numbers and what I ate. For me it was a great diary.’	Patients are motivated to improve self-management because they know their provider is watching. ‘Sometimes I forget to take my blood glucose, but if I knew that someone was looking at them, then I will be more compliant.’ ‘If I knew that someone was looking at this information on the other end at BMG, I would definitely continue to use it.’ ‘Just physician acknowledgement is very useful’
8. Patients’ experiences of self-monitoring blood pressure and self-titration of medication; Miren I Jones, Sheila M Greenfield, Emma P Bray, Sabrina Baral-Grant, FD Richard Hobbs, Roger Holder, Paul Little, Jonathan Mant, Satnam K Virdee, Bryan Williams and Richard J McManus	Twenty-three patients and 6 family members; UK; to explore patients’ views of self-monitoring blood pressure and self-titration of antihypertensive medication	Patients were confident about self-monitoring	Yes	Additional support needed for some patients. Although patients received training on making medication changes without seeing a GP, several chose to reconsult before implementing such changes. This was usually because they lacked confidence or because of problems with their medication-change forms. Family members of patients who needed assistance with the trial were more cautious about implementing changes, and relatives of patients 25 and 30 accompanied the patient to visit the GP before making changes during the introduction but were confident to continue alone if had input at this early stage additional support or tailoring for some groups of patients needed; generic input by network members might be insufficient	Blood pressure monitoring Patients felt that home blood pressure readings were more ‘natural’ than surgery readings, as they were more relaxed at home and the readings were taken more carefully and under controlled conditions Not about fit with existing practice, but perceived improvement; adding/enhancing what is perceived as ‘best conditions’?	Many were surprised at how much their readings varied, and some suggested that changing their medication based on fewer readings was not appropriate, due to this variability. This led them to question whether a GP should adjust a patient’s medication after taking a single reading: ‘I was amazed how much they varied. That was very educational. I mean okay if there’s a crisis or something, you expect your blood pressure to go up, but I could take them just sitting there and it was just amazing the difference in them.’ (P29, F, 78, made medication change 1, medication change 2 postponed)
9. Innovation and evaluation: taming and unleashing telecare technology; Jeannette Pols and Dick Willems	Nine COPD patients; Holland; Telecare is advocated in most European countries with great, if not grandiose, promises: improving healthcare, lowering costs, solving workforce shortage. This paper does not so much question these specific promises but rather the ‘register of promising’ as such by comparing the promises with actual processes of incorporating technologies in healthcare practices	In order to function, at all, technology has to be tamed, it has to be tinkered with to fit the practices of the users. The technology, however, is not meekly put to use (tamed), but is unleashed as well, affecting care practices in unforeseen ways. The untenability of pre-given promises and the fluidity of locally evolving goals has important implications for the way in which innovations are promoted, as well as for the way innovative technologies may be evaluated.	Yes	Rather than policy notions of ‘dealing with ageing population’ and ‘reducing caregiver-patient visits’ the clinicians concerned formulated goals that were relevant for their own practices: to deliver good care and improve care if possible. Support and company from other participants ‘Oh yes, seeing each other [over the webcam] is different from talking on the telephone… It is much more personal. And much cosier! For instance with Peter’s wife, when I talk to Peter she comes along to chat for a bit. And she was there [at the clinic] a lot too, same as my husband. And he chats along too. Or the guys chat together: “Gosh, how are you”, or; “We are in town this Saturday, will you be home?”’	The video conversation was experienced as much more personal and intense than telephone conversation rather than resembling the ‘real encounter’. Telecare was made interesting to the users as a necessary step for users to put in effort and make it work. Attempts to extend the use of the telekit to a wide range of users did not work and there was a high level of dropout. This was because: there was not enough space in the house, not wanting to have an extra computer, plans for moving house, not wanting to make the disease too central to their lives. Also for people who were in better health and went back to work telephone calls were disruptive and using the telekit was not practical. The telekit was based on Apple application and this created problems of usability as most people were familiar and/or had access to windows and thus had to later adapt their knowledge; the volunteers providing the training also did this on windows. The telekit obtained two different identities that were in conflict: guaranteeing the effect of the treatment and providing a window to the world. These required different ways of ‘taming’ the technology. The hardware was available for 3 months only and after that only training was given, but it was not seen as the responsibility of the clinic to provide computers access beyond this point. The use of the internet and e-mail had the potential of becoming structural activities, as ways of seeking entertainment and keeping contacts. Using the internet could support services such as having the shopping brought to one’s home, becoming a member of online entertainment sites, for example, to play bridge, or joining social websites. The telekit would thus become more structurally part of the social, practical and emotional life of the patients, allowing for many different contacts, after the carer from the clinic had stopped seeing them. It would be a device to help patients build up their new world. Brings structure to a disrupted everyday life The participation in The day’s start may in this sense be an aid to structure the day, by getting up in the morning in time to attend. This is what one of the patients mentioned. After his discharge from the clinic, back at home he felt he dropped into a black hole. The telekit helped him to structure his days Opening new possibilities unused before ‘We teach them to write e-mails. And there was one man, he had a son who lives in Japan. And in the meantime he has become a grandparent, but he had never heard of the internet. So he got this internet connection at home, and his son sent him his email address. And I helped him typing the e-mail address, and when he got an answer he got pictures and saw his grandchild for the first time. Really, if you see this older man looking at a picture with tears in his eyes’	Continuity of care and the telekit as an ‘umbilical cord’ ‘Ah, over the webcam you can see the smoking cigarettes in the background. When someone says: I am doing fine! You can see from the way somebody keeps his or her body that they are not fine at all. When you can see that, you can say: your shoulders are too high, are you ok?’ Contacts with fellow patients would serve the same purpose of bridging the gap between the clinic and the home
10. Dismantling sociocultural barriers to eye care with tele-ophthalmology: lessons from an Alberta Cree community Sourabh Arora; Ayaz K Kurji; Matthew TS Tennant	Aboriginal patients, cultural liaison, nurses and program administrators; Canada; to determine whether tele-ophthalmology services, provided to Aboriginal Canadians in a culturally sensitive community-based clinic, could overcome social and cultural barriers in ways that would be difficult in the traditional hospital-based setting	The introduction of culturally sensitive programs led to increased appointment attendance; from 25% to 85%. Involvement of Aboriginal nurses, inclusion of culturally sensitive activities and participation in spiritual ceremonies led to qualitative accounts of increased patient satisfaction, trust towards the healthcare team and communication amongst participants	Yes	When the nurse would speak to patient she would ask ‘how do you feel emotionally?’ and ‘how do you feel spiritually?’ patients felt that when vision is lost, it was also spiritual loss and aborigional nurses could understand this better	The delivery of the programme was made to resemble practices familiar from encounters with traditional healers. This included provided snacks as traditional healers would do, ‘smudge’ ceremony, sitting in a circle and discussing health together with spiritual and emotional health as aspects of adopting healthy living, setting a tepee outside the centre where the programme was delivered. This was supported by an aboriginal nurse and/or a liaison assistant. There were financial, transport and distance barriers to attending	
11. Attitudes to COPD patients towards tele-rehabilitation: a cross-sector case study; Birthe Dinesen, Lotte Huniche, Egon Toft	Twenty-two COPD patients; Denmark; to describe patients’ attitudes towards tele-rehabilitation in the Danish TELEKAT to better understand patients’ behaviour when performing tele-rehabilitation activities in home surroundings	COPD patients exhibit four types of attitudes about their tele-rehabilitation: indifference, learning as part of situations in everyday life, feeling of security and motivation for performing physical training. The patients express the view that they circulate between these attitudes depending on their physical and emotional state as they perform their training. The COPD patients and healthcare professionals have created a community of tele-rehabilitation across sectors, exchanging experiences, stories and strategies for how to manage rehabilitation in home surroundings	Yes	Sense of common interest and purpose The healthcare professionals experienced the patients as having a common interest in tele-rehabilitation and in participating directly in their rehabilitation. Active involvement by healthcare professionals The healthcare professionals reported that they gave some of the patients more responsibility for managing their own disease, and the patients received a treatment plan consisting of prescriptions for penicillin and hormones and guidelines for what to do in case symptoms appeared. In this way, the patients became more active, changing their mind-set, and were able to perform self-management of their COPD. Need for tailoring of level of professional and network involvement Family involvement and support The patients reported that family and network became more engaged in the tele-rehabilitation program of helping the patient to integrate the activities into their everyday routine and maintain the focus on exercise as a normal part of their everyday lives. Felt supported and expected further monitoring Those patients who were using oxygen in their homes felt that the 16-week tele-rehabilitation period was too short. They preferred the possibility of being monitored permanently. encouraged by having access to remotely supervised feedback from healthcare professionals; interaction with healthcare professionals and other patients encouraged them to carry out their physical training at home and to push themselves. The intervention as a process of changing relationships within wider network The interaction between the COPD patients and healthcare professionals in the tele-rehabilitation programme can be characterized in terms of Wenger’s approach as a ‘community of tele-rehabilitation’. This community links COPD patients, their family members and healthcare professionals across sectors. The COPD patients have expressed the view that their relationships with the healthcare professionals had developed from that of being subordinated to professional authority to a relationship of dialogue, where the focus was on mutual learning NOTE: the web platform also enabled discussions with other users, but this is not discussed in the paper; not clear how much this was used; relationships change discussed primarily in relation to professionals and existing network members	Good fit with time and space These 17 patients expressed the view that the technological platform in the TELEKAT project opened the possibility for them to obtain data, share data and communicate with healthcare professionals and other patients independently of time and space. Fit with daily life These 12 patients found that the tele-rehabilitation programme gave them time to try new exercises, to become more involved, and to adjust their training program to their home environment and situations in everyday life. They found it convenient that they could do their exercises at home, at any time. Access Easy access to healthcare professionals Changing needs and attitudes to tele-rehabilitation; flexibility and telehealth as a process COPD patients exhibit four attitudes about their tele-rehabilitation: indifference, learning as part of situations in everyday life, feeling of security and motivation to perform physical training. The patients express the view that they alternate between these attitudes, depending on their physical and emotional state as they perform their rehabilitation exercises	Gained new knowledge Twelve patients reported that they gained new knowledge by communicating and interacting about the measured values, symptoms, medication and exercises, as well as in the social and cultural process of exchanging experiences, stories from everyday life and how to address rehabilitation-related issues in their home surroundings. Visibility of measurements and awareness of symptoms The patients stated that they became more aware and reflected upon the measured values and symptoms in their COPD. Via their interaction with the healthcare professionals and other patients in the programme, they learned to become more aware of their own symptoms and to know when it was necessary to contact a doctor at an early stage in order to seek treatment. As one patient said: ‘Seeing my data on the web portal gives me a better understanding of how to exercise and interpret the development of my symptoms when I experience the onset of an exacerbation.’ Being able to actually see the graphically presented data (blood pressure, pulse, weight, spiometry, and saturation) on the web portal or tele-health monitor motivated the patients to continue training and to compete with themselves, especially when the measured values showed improvement over time. Observed no effect of intervention A small number of patients (5/22) experienced indifference towards the tele-rehabilitation measures. The patients argued that it was because the measured values (blood pressure, pulse, weight, spirometry, and saturation) were stable. These patients reported that they were unable to observe any connection between measured values and physical training over time when they followed their data on the TELEKAT web portal or on their tele-health monitor. Demanded further monitoring Those patients who were using oxygen in their homes felt that the 16-week tele-rehabilitation period was too short. They preferred the possibility of being monitored permanently
12. Telemonitoring for chronic heart failure: the views of patients and healthcare professionals—a qualitative study; Peter Fairbrother, Jenny Ure, Janet Hanley, Lucy McCloughan, Martin Denvir, Aziz Sheikh and Brian McKinstry on behalf of the Telescot programme team	Eighteen patients, 5 professionals; Scotland; to understand the views of patients and professionals on the acceptability and perceived usefulness of telemonitoring in the management of chronic heart failure in the context of day-to-day care provision	Telemonitoring was popular with patients because they felt reassurance arising from what was perceived as continuous practitioner surveillance. Professionals expressed concern regarding perceived patient dependence on practitioner support. Increased workload was also a concern	Yes	Support and reassurance Many stated that they liked being telemonitored because they felt reassurance arising from what was perceived as the provision of continuous practitioner surveillance and support Depth and frequency of communication and growing dependence Many professionals considered that patients’ access to telemonitoring data combined with increased accessibility of healthcare professionals operating telemonitoring services increased both the depth and frequency of communication between patients and professionals. While this was often considered a good thing in terms of supporting early intervention and preventing deterioration in health, professionals also expressed concern regarding perceived greater patient dependence on practitioner support. Tensions between patient and practitioner interpretations Many thought the service was designed to increase practitioner support rather than to foster greater personal responsibility: ‘I know if there is something wrong, they are going to pick it up right away… if something goes wrong, they’ll phone me. [It’s a] safety net.’ (patient #29: male, 79 years old) Practitioner attempts to encourage involvement in self-management (for example, in attempts to encourage patient participation in self-directed medication) received a mixed response, with some expressing anxiety and trepidation at the prospect of being required to exercise greater personal responsibility Tension between objectives: monitoring and SMS Professionals queried the utility of the telemonitoring technology in supporting patient self-management. One respondent remarked that the IHG questionnaire was devoid of questions or prompts to encourage and support self-management attitudes or behaviours. Relationships based on continuity of care Many patients expressed a preference for being telemonitored by professionals with whom they had an existing association. Professionals discussed proposed models of future telemetric provision, including the proposition of a centralised regional service, operated by nonclinical professionals, which had been mooted by healthcare managers. They considered integration of telemetric provision with local practitioner services preferable to centralised ‘call centre’-type provision, emphasising the value of relationship-based continuity of care over cost benefits associated with centralisation	Usability Telemonitoring was extremely popular with patients. All of the respondents found the technology easy to use. Both groups described numerous technical difficulties with the technology, teething difficulties, concerns and design issues. Patients and professionals reported experiencing technical problems with the equipment, notably recurrent malfunctions with the peripheral devices. Some commented on the intrusiveness of the equipment, remarking on the noise and luminosity of the IHG and its size or bulkiness in the home. Cost of equipment and maintenance Both groups raised concern regarding the expensiveness of the equipment, the cost consequences of installation (which required the fitting of broadband cabling in patients’ homes) and the ongoing costs of support and maintenance Integration with existing systems Professionals described problems arising from the perceived lack of interoperability between the (stand-alone) telemonitoring patient information system and existing patient information systems used in both primary and secondary care. They indicated frustration with the limited functionality of the telemonitoring system, and the compartmentalisation of data between telemetric and usual care information systems. Disruption of established medication regime Changes in medication were not always received with enthusiasm by patients. Some felt that alterations to their medication did not result in an improvement in their condition. Such changes led some to query prescribing practice under usual care: ‘As a result of [telemonitoring], they increased the quantity of one of the drugs I’m taking… which hasn’t made the slightest difference.’ (patient #22: male, 76 years old) Workload and practice The impact of telemonitoring on home visits and existing practice was of particular concern to professionals. The telemonitoring responsibilities undertaken by professionals were additional to existing professional responsibilities. Consequently, professionals expressed the view that telemonitoring added to workload. They considered it time and resource intensive, describing the work involved in checking online data, in dealing with additional administration, and in increased communication and interaction with patients	Feedback and new knowledge Patients also expressed the view that they felt better informed and more knowledgeable about their condition as a result of involvement in the telemonitoring. Many found it helpful to know their weight, blood pressure, and oxygen saturation score and to have the facility to monitor data trends over time. This was considered beneficial in determining state of health: ‘It keeps you in the picture… And you know exactly what’s going on from day to day… And it also lets [the telemonitoring nurse] know exactly what’s going on*…*’ (patient #2: male, 75 years old) ‘I felt quite happy to be involved… instead of just being a vegetable that sat back and swallowed things.’ (patient #24: female, 79 years old) For professionals: facilitated closer monitoring Professionals perceived that telemonitoring facilitated ‘closer monitoring’ of patients. Telemonitoring data were attributed as providing a more detailed picture of patient health than usual care, enabling the professional to take pro-active approaches to clinical management Improved prescribing For some professionals, telemonitoring supported the development of prescribing practice, providing an evidence base for the trialling of medications on selected patients.
13. Spanning boundaries into remote communities: an exploration of experiences with telehealth chronic disease self-management programs in rural Northern Ontario, Canada; Sara J.T. Guilcher, Tarik Bereket, Jennifer Voth, Vinita A. Haroun, Susan B. Jaglal,	Forty-four participated in focus groups; Canada;to explore the experiences of participants in a chronic disease self-management program via telehealth (tele-CDSMP) and to identify facilitators and barriers to inform future tele-CDSMP delivery models in rural and remote settings	Four main themes were identified by tele-CDSMP participants related to the overall experience of the program: (1) bridging the access gap, (2) importance of group dynamics, (3) importance of strong leaders, and (4) preference for extended session time. Key barriers were related to transportation, lack of session time, and access to Internet-based resources. The main facilitators were having strong program leaders, encouraging the development of group identity, and providing enough time to be comfortable with technology	Yes	There could be stigmatization about CI in their everyday life and the group provide a safe and supportive environment. The program encouraged building new connections and avoiding isolation. Some of the people remained in contact after the end of the programme. Developing accountability and motivation via peer coaching. Support by peers extended beyond the meeting site and continued outside of the studio. Success of group depended on having supportive and knowledgeable group leaders, who created good group dynamic. Insufficient time to discuss action plans and concerns one to one with the facilitator, which was available in face to face sessions but not in the telehealth intervention	Participants were hesitant at times to speak into the microphone and participate, and effective facilitation was important in overcoming that. While the group dynamic worked it took a couple of sessions before the participants felt comfortable with the technology. Access to Internet-based resources was mentioned as one of the barriers for participation. Transport to site of teleconferencing a barrier	The group provided a mechanism for sharing experiences and feelings about CI. The group environment encouraged information sharing about community resources and individual strategies to encourage day-to-day healthy eating, nutrition, and exercises
14. Piloting tele-monitoring in COPD: a mixed methods exploration of issues in design and implementation; Jenny Ure, Hilary Pinnock, Janet Hanley, Gillian Kidd, Emily McCall Smith, Alex Tarling, Claudia Pagliari, Aziz Sheikh, William MacNee, Brian McKinstry	Twenty of the 27 patients in the pilot and 25 professionals participated. (*n* = 55 interviews and one focus group); Scotland; to explore the perceptions of patients and professionals about the pilot implementation of the COPD tele-monitoring service	Tele-monitoring was perceived by patients as improving access to professional care, but raised concerns for clinicians about possible over-treatment and how best to organise services to support the technology	Yes	Some patients appreciated the possibility of a remote consultation informed by the tele-monitoring data, thus avoiding the need to travel to the surgery. ‘You know if something was wrong I’d get a phone call from the surgery… they’d write a prescription and I’d get it sent to the chemist and then I’d get it delivered direct. Because if I’m unwell that’s one thing I have to face is that long walk to [the surgery], because there’s no bus direct from here and, you know, when I’m unwell’ (Female patient, 66 years old, post-installation) Empowering self-care or increasing dependence? There was an over-riding sense from patients and carers that the tele-monitoring system ‘watched over’ and ‘looked after’ them. Although some patients seemed to be describing an abrogation of personal responsibility as the technology could take over the decision about whether action was needed, most perceived that having access to readings and emergency supplies of antibiotics at home gave them confidence to respond to deteriorating symptoms themselves. ‘In a way it was a relief thinking that I should ignore my own thoughts on getting a doctor or something like that. This organisation was going to get hold of a doctor if their readings showed I needed a doctor.’ (Female patient, 47 years old, post-installation)	Usability Despite some irritations with the technology, patients were generally very positive about the tele-monitoring service. Completing the symptom scores was not always intuitive for patients who were constantly symptomatic. The questions asked if breathlessness or cough had increased, but some patients found it difficult to set a standard against which to benchmark their symptoms. ‘It asks “Is it higher than normal?” I don’t know what the normal’s supposed to be. So I don’t know what… Sometimes it’s not all black and white.’ (Male patient, 69 years old, post-installation). In general, the professionals’ perception of benefits outweighed the initial rearrangement of work practices to accommodate the monitoring. Increased workload Workload had increased as telephone calls or, occasionally, visits were made to clarify the reality of the individual clinical situation when the score breached the threshold. At the observation visits it was noted that the equipment generally integrated well into the home environment, although the size and background noise of the computer fan caused some problems in smaller more crowded living conditions. Most users quickly became familiar with the technology and found it easy to use. One particular worry was that the system failed to confirm that data had been transmitted, leaving patients uncertain whether the monitoring had been successful. None of the patients were concerned about confidentiality - if anything, they were worried that their data may not be shared widely enough	Patients consistently expressed anxieties about managing exacerbations, describing the difficulty of recognising the onset of an exacerbation, delays as they considered whether to seek professional advice, and the practical barriers to accessing professional care. Tele-monitoring was almost universally considered by both patients and their carers as helping to address these problems ‘You’d think you would find it easy to tell when you’re ill but it’s only afterwards that you know you are not well. But this technology is really brilliant.’ (Female patient, 66 years old, post-installation) Recognising exacerbations or over-treatment? In keeping with the patients’ assessment that the telemonitoring was sensitive to changes in their condition, clinicians initially assumed that the marked increase in prescriptions for antibiotics and steroids reflected improved recognition of exacerbations, although the possibility that the threshold for triggering an alert may have been set too low was raised. Later, the risks and benefits of what was perceived as a substantial increase in prescribing became a concern.
15. Perceptions of successful cues to action and opportunities to augment behaviour triggers in diabetes self-management: qualitative analysis of mobile intervention for low income Latinos with diabetes; Burner ER, Menchine MD, Kubicek K, Robles M, Arora S	Five focus groups with 24 people; USA; we examined nuances of motivation, intention, and triggers to action effected by TExT-MED (trial to examine text messaging for emergency department patient with diabetes), an mHealth intervention tailored to low-income, urban Latinos with diabetes	Low-income Latino patients will accept text messages as a behavioural intervention. This mHealth intervention acts as a behavioural trigger rather than an education platform. Personalization is an opportunity to enhance these cues to action	Yes	Recommended programme to family and friends The appearance of support, although aware this is an automated service Participants said they would feel more cared for if the messages were personalised, including the name and the specific time for taking medication (programme used the same messages across participants to increase scalability). Participants wanted personalisation in terms of negative consequences of the disease specific to the stage at which each one of them was	Found behaviour cues and medication reminders easy to follow	Sense/perception that the programme has worked for them to take control over diabetes and make the behaviour changes they believed to be of benefit for them ‘better controlling our life, our way of living’. Medication reminders were the most salient and prominent benefits mentioned. This included specific prompts. Healthy living challenge messages about specific behaviours had an impact; ‘the challenges you send us. One imagines that when I see the message, and when I read the challenge, those are my challenge for the day…I read them and say, “I have to do this”. I motivate myself, like if I am going to go for a walk.’

## Results and discussion

### Relationships

The importance of relationships with professionals for mediating the introduction and uptake of self-care support has been noted in long-term condition and self-management literature and refers to elements that professionals bring to a therapeutic approach with patients. Empathy, acceptance, and the therapeutic alliance between therapist and client are considered essential to effective psychological therapies [26] and involvement of a health professional to provide legitimization of strategies for self-management [27]. We examined the literature to see why and how relationships were important for the success of a telehealth intervention, exploring the contexts, aspects of relationality (for example, continuity, communication, and rapport), differences between peer and patient-professional relationships, and whether telehealth technology augmented or substituted for face to face/personal contact.

### Evidence about telehealth reshaping existing relationships: open ended, contextually embedded, dependent on additional support

Telehealth interventions are sometimes framed in terms of a threat to professionals and as leading to a deterioration of previously valued relationships and roles. Segar et al*.* [28] note a sense of protectiveness about maintaining boundaries around established remits of managing long-term conditions when new technologies are introduced. However, the presumption that technology is a threat to existing relationships when it comes to the *actual* use of specific technologies for self-management is not borne out by patient accounts in the studies we reviewed. Moreover, the introduction of new technologies establishes new sources of relationality and is therefore better viewed as restructuring existing relationships in a process that is open rather than leading to a pre-determined outcome. For example, the frequency of contact and approachability of professionals engaged in telemonitoring led to building new bonds of trust and introduced an intermediary function between patient and GP.

‘I’d say you get better [service] because if [telemonitoring professional’s first name] comes on the phone and she’ll say “I think you’re needing to speak to the doctor”, she’s just giving me a warning that she’s going to get the doctor to phone me. And they’ll either say “Well, I think you’re needing to have some antibiotics” or “I think maybe we should pop over and just see you.’ [29]

Not only can technology build trust in the absence of pre-existing relationships, the introduction of technology can potentially offer more control for users through the introduction of distanced but positively perceived relationality. For example, self-management support (SMS) was preferred to a telephone conversation because it provided more control over managing relationships.

‘I liked the project and the follow-up. I could send an SMS whenever I wanted. I got an answer within half an hour. I especially liked the SMS - in the Netherlands, where I lived prior to this, I knew I could call, but I like the message system a lot better’ [30].

The indeterminacy as to *how* telehealth interventions can reshape existing relationships is evident in relation to their impact on the division of labour between users and professionals through increasing the input of users, professionals, or network members, or changes through building a shared network of responsibility. For example, Fairbrother et al*.* [29] report that practitioners’ attempts to encourage patient participation in self-directed medication received a mixed response, with some expressing anxiety and trepidation at the prospect of being required to exercise greater personal responsibility. Many users stated that they liked being telemonitored because they felt reassurance arising from what was perceived as the provision of continuous practitioner surveillance and support. By contrast, telehealth interventions can lead to more active engagement by users, professionals, and network members which allows for the better tailoring of illness management. In a study by Dinesen et al*.* [31], healthcare professionals reported that a tele-rehabilitation programme allowed the handing over of responsibility for managing their illness at the same time patients reported that family and network members became more engaged in helping them to integrate the activities into their everyday routine and to maintain the focus on exercise as a normal part of their everyday lives.

### Telehealth as a substitute to the role of health professionals: legitimising and strategic role

There was evidence, in our sample, to support the notion that telehealth *can* work without professional input. There was also evidence that telehealth interventions with *no* professional input were more likely to be successful if they were individually tailored: that is, including the name of the user, the specific time for taking medication, and the negative consequences of the disease specific to the stage at which each one of them was [32].

Cases where telehealth intervention could work in the absence of any professional input were rare in the papers we reviewed [18]. Thus, while professional relationships did not appear to always be essential they could improve acceptability and outcomes even where this professional input was minimal for example facilitating or linking people to using telehealth. Professional ‘lite’ input might also work through a process of users feeling reassured or construing professional monitoring as part of a partnership where responsibility is shared.

‘In a way it was a relief thinking that I should ignore my own thoughts on getting a doctor or something like that. This organisation was going to get hold of a doctor if their readings showed I needed a doctor.’ (Female patient, 47 years old, post-installation) [33].

‘If I knew that someone was looking at this information on the other end at BMG, I would definitely continue to use it.’ [34]

### The role of relationships derived from peer support: need for tailoring and extending existing networks

There was limited evidence to support the necessity of peer relationships as the defining ingredient of the success of an intervention. The presence of generic peer support was likely to be insufficient and implicated the need for tailoring to different needs and circumstances. Tailoring related to age and gender appears to be the most salient features of interventions. However users are likely to feel more comfortable if they have control over the technology, how it is used, and the circumstances under which they might disclose (or not) their involvement with a telehealth programme, thus avoiding potential embarrassment: ‘if this popped up, I think people would laugh at me’ [31].

Interventions that opened up opportunities for extending existing networks seemed likely to be successful and related to the inherent capacity of interventions for flexibility in support in relation to context and individual needs. Some newer social media - for example, those using video - offered richer contextual awareness enhancing people’s support networks and contributing to successful interventions [35].

‘Oh yes, seeing each other [over the webcam] is different from talking on the telephone. It is much more personal. And much cosier! For instance with Peter’s wife, when I talk to Peter she comes along to chat for a bit. And she was there [at the clinic] a lot too, same as my husband. And he chats along too. Or the guys chat together: ‘Gosh, how are you’, or; ‘We are in town this Saturday, will you be home?’ [35]

‘Ah, over the webcam you can see the smoking cigarettes in the background. When someone says: I am doing fine! You can see from the way somebody keeps his or her body that they are not fine at all. When you can see that, you can say: your shoulders are too high, are you ok?’ [35]

Possibilities for extending networks are likely to be of high value in contexts where members of one’s intimate network, such as family, might act as a barrier by being too cautious about making changes to aspects of illness management (for example, medication) [31]. Additionally, in a programme delivered to rural communities, Guilcher et al*.* [36] found that in a context of stigma the programme provided a safe and supportive environment, encouraged building new connections and avoiding isolation. The success of the programme was dependent on support by peers extending beyond the meeting site and on having supportive and knowledgeable group leaders, who created good group dynamic.

### Fit

When we examined the literature, we found that the extent to which a telehealth intervention worked was in part dependent on the extent to which there was a fit with patients’ needs, skills, and daily life. In addition to the importance of fit, we also found that context played a facilitative role in providing the necessary conditions for workability. For example, home blood pressure readings were evaluated by users as being more ‘natural’ than surgery readings (and therefore more accurate), as people were reportedly more relaxed in domestic settings and the readings were taken more carefully and under controlled conditions [37].

### Fit with patient-defined needs and environment

There was evidence that interventions which were perceived as ‘fixing’ a problem from the patient’s point-of-view might fare better than others which did not. For example, people with COPD found a tele-rehabilitation programme convenient as they could do their exercises at home at any time, could try new exercises, become more involved in the management of their illness, and could adjust the training program to their home environment and situations in their everyday life [31,38]. When successfully adapted to people’s needs and environment, telehealth interventions had the capacity to enhance accessibility of health care for those who might otherwise not access traditional face-to-face care or those who were geographically isolated living in remote areas with poor transport options.

‘You know if something was wrong I’d get a phone call from the surgery… they’d write a prescription and I’d get it sent to the chemist and then I’d get it delivered direct. Because if I’m unwell that’s one thing I have to face is that long walk to [the surgery], because there’s no bus direct from here and, you know, when I’m unwell’ (Female patient, 66 years old., post-installation) [39].

The use of telehealth could also offer convenience in accessing care opening up the possibility to obtain and share data and communicate with healthcare professionals and other patients independently of time and space [31]. Fit with needs and context can be seen as an ongoing and reflexive process. Continued utilisation is likely to be constant review and dependent on users continuing sense of added value.

‘If it gets it wrong, you would automatically get really irritated by it…I think the risk if getting it wrong would be really annoying and I’d probably delete the app’

‘It’s quite easy to lose interest really because it is quite an effort and nobody wants to spend all their life writing down what they want on their phone’ [40].

There was also evidence that interventions were likely to be unsuccessful where users found them disruptive and impractical. This was the case for people in better health who went back to work, had plans to move house, did not have enough space in the house to accommodate the telecare equipment, or did not want to make the illness too central to their lives [35]. Other barriers to using telehealth were described by professionals in relation to limited functionality and the lack of interoperability between telemonitoring patient information systems and the existing systems used [29]. Patients also require time before they become comfortable with using technology, and experienced problems in accessing internet, and getting transport to the site of the teleconference [35,39].

### Fit with patient skills and capacity

There was evidence that patients’ capability in technology use increased their propensity to benefit from interventions. Simple technologies appeared effective suggesting that technologies that are already used routinely in everyday life may be easier to use to deliver telehealth. This is most clearly illustrated when delivering simple and easy-to-follow messages such as medication reminders or specific prompts such as healthy living challenge messages.

‘the challenges you send us. One imagines that when I see the message, and when I read the challenge, those are my challenge for the day…I read them and say, I have to do this’. I motivate myself, like if I am going to go for a walk.’ [32].

Telehealth interventions may require some level of basic training and need to be tailored to existing user skills and physical capacity.

‘If this is for people like me, there should be adjustments for functional limitations. Here they talk mainly about the possibilities, about people who are mobile etc., but the people who cannot get out of the house, for those adjustments should be made.’ [41].

Engaging with technological interventions could provide new possibilities for learning through enhancing an existing skill set.

‘We teach them to write e-mails. And there was one man he had a son who lives in Japan. And in the meantime he has become a grandparent, but he had never heard of the internet. So he got this internet connection at home, and his son sent him his email address. And I helped him typing the e-mail address, and when he got an answer he got pictures and saw his grandchild for the first time. Really, if you see this older man looking at a picture with tears in his eyes’ [35].

### Fit with the structure of daily life

The appeal of a programme might be enhanced through embedding it into a set of familiar relationships and cultural practices. This was for both professionals and users. For example, the uptake of tele-ophthalmology by an aboriginal community improved when its delivery was made to resemble practices familiar from encounters with traditional healers and supported by an aboriginal nurse and/or a liaison assistant [42]. Professionals expressed the view that telemonitoring added to workload as it required additional time to checking online data, dealing with additional administration, and increased communication and interaction with patients [33,39]. These findings were consistent with the wider literature on professional involvement [43].

The notion of ‘fit’ operates within an assumption about the relative structuredness of everyday life and a value attached to it. There are however, situations and contexts where this might not be the case. There is evidence of how the use of new technology brings a new structure to disrupted everyday lives. For example, telecare can aid re-structuring the discharge from other care services, for example, discharge from clinics can mean potential disorientation when back in domestic settings. This can be potentially averted by the deployment of ‘telekit’ which helps to structure the day [35] and to ‘better controlling our life, our way of living’ [44]. Technology can also help adapt to changing needs and attitudes to tele-rehabilitation. Ongoing sustainability of use requires not only about access to and investment in technologies - for example, broadband upgrades and knowledge of new ‘apps’ [33], but also a level of interest in making telehealth work [40].

Motives for engaging, learning, and enacting new skills fade when there is ongoing stability in measurements or if the patient is constantly symptomatic (this is a particular problem in COPD). Adjustment and interest is made possible if technology use can be extended to incorporate other network members, for example, Dinesen et al*.* [31] reported that family and network became more engaged in a tele-rehabilitation program of activities and helped the patient to maintain the focus and integrate the activities into their everyday routines.

### Visibility

We identified earlier that telemonitoring of symptoms and vital signs were perceived by patients to have positive impact on outcomes. This led us to consider that this might have reinforcing and incentivising functions (for example, reporting vital signs encouraged self-regulation, or the belief that healthcare professionals were monitoring information encouraged patients to follow instructions, or enabled healthcare professionals to respond to patients’ needs quickly). Visibility is likely to work best for some physical conditions and diseases - diabetes, heart failure - more than others. This ‘making visible’ to self or others seemed to have a powerful role in empowering users and opening up possibilities for engagement with their network members. Indeed, the surveillance aspect of technology was experienced as a reassurance of continuous practitioner engagement and support [33]. Nevertheless, visibility was not always welcome because some patients were worried about data getting into the hands of a third party and found some phone capabilities intrusive [40].

### Visibility linked to knowledge, motivation, and empowerment

The use of glucose-monitoring web application improved visualisation of blood sugar profiles and increased patients’ general awareness of their blood sugar and its changes.

‘Graphs are a quick way of seeing how [blood sugar] fluctuates.’

‘I found it much easier to keep track of both my numbers and what I ate. For me it was a great diary.’ ‘Found it kind of helpful and fun to track what I was eating and how that affected my readings… in particular when I was supposed to have a medication change’ [34].

Fairbrother et al*.* [38] reported that users found it helpful to know their oxygen saturation, to learn their ‘normal’ range by identifying telemonitoring data trends over time, and linked indicators to level of physical effort and the onset of vital signs [31].

‘I’m okay from 87% (oxygen saturation) upwards and I never get better than 92. Even when I’m very well, I never get better than 92. But I go out and about and I do what I need to do and I manage it by walking’ [38]

‘Seeing my data on the web portal gives me a better understanding of how to exercise and interpret the development of my symptoms when I experience the onset of an exacerbation.’ [31]

There was some evidence that involving patients in monitoring promoted feelings of empowerment and made users feel more knowledgeable, but it also gave them a sense of being actively involved in the management of their illness.

‘It keeps you in the picture… And you know exactly what’s going on from day to day… And it also lets [the telemonitoring nurse] know exactly what’s going on…’

‘I felt quite happy to be involved… instead of just being a vegetable that sat back and swallowed things.’ [33]

Visible reminders encouraged participation and motivated patients to continue training and competing with themselves especially when values improved over time [31]. Telemonitoring helped patients and carers to recognise the onset of an exacerbation and allowed them to better address these problems.

‘You’d think you would find it easy to tell when you’re ill but it’s only afterwards that you know you are not well. But this technology is really brilliant.’ (Female patient, 66 years old, post-installation) [39].

By contrast, patients whose measured values (blood pressure, pulse, weight, spirometry, and saturation) were stable over time, and thus were unable to observe any connection between measured values and physical training, were indifferent towards the intervention [31]. Patients with COPD who were constantly symptomatic, and thus with no standard against which to benchmark their symptoms, found a telemonitoring intervention lack utility.

‘It asks “Is it higher than normal?” I don’t know what the normal’s supposed to be. So I don’t know what … Sometimes it’s not all black and white.’ (Male patient, 69 years old, post-installation) [39].

### Visibility linked to the engagement of others

Systems which encouraged accountability such as an expectation that patients would check their blood glucose levels frequently, coupled with feedback from the healthcare staff facilitated through technology, also worked well. For example, diabetes patients reported that ‘when you have a date [upcoming videoconference] you are more likely to do something’ [9]. Support by peers or professionals also enhanced visibility and perceived personal accountability. For example, through interaction with healthcare professionals and other users of the programme, patients with COPD became more aware of symptoms, learned to understanding measured values, and became aware when it was necessary to contact a doctor and seek treatment [31]*.*

Visibility also led to user learning and restructuring of relationship with professionals. Jones et al*.* [37] reported that many users were surprised at how much their readings varied, which led them to question whether a GP should adjust their medications only after taking a single reading:

‘I was amazed how much they varied. That was very educational. I mean okay if there’s a crisis or something, you expect your blood pressure to go up, but I could take them just sitting there and it was just amazing the difference in them.’ [37]

## Discussion

In a rapidly developing field where a range of influences relating to the uptake technologies have been associated with discrete telehealth and telecare technologies, this review extended and deepened our understanding of the likely core and generic mechanisms underpinning successful interventions. These are important to identify in order that new designs can take account of this upfront and from the outset of development. The realist synthesis offered three possible mechanisms that make interventions ‘work’. Stage 2 review process tested these, confirming them and allowing us to enhance our conceptualisation. In terms of ‘relationships’, the evidence suggested two processes which enable successful telehealth interventions: relationships provide *support* (professional, peer, clinical, and social) for behaviour change, and relationships provide opportunities for professional feedback which *reinforces* positive or required behaviour change. This suggests that interventions which enable connections and contact, notably between patients and professionals can facilitate support and reinforcement necessary for behaviour change. Where telehealth interventions limit or remove the relationship between patients and professional, other opportunities to support and reinforce behaviour may be necessary. In terms of ‘fit’, the literature pointed to the importance of acceptability and ease of use of telehealth interventions for patients and professionals. Telehealth can increase *accessibility of care* for some populations. *Simple* technologies appeared to work as well or better than more complex ones, and there are some patient groups who are less able to use some technologies (notably the Web) [11,45,46]. Fit also relates to the extent to which technologies disrupt existing environments and ways of managing. The third proposition centred on ‘visibility’, which is how telehealth care makes an illness or condition apparent to the self and others. There is a connection between the visibility that technologies promote and the capacity to self-manage. Visibility brings enhanced opportunities to share and engage information and tasks with others in a person’s network. Visibility facilitates and mediates knowledge and motivations which are inextricably linked to the actual tasks of self-management. The evidence suggested that visibility operated by enabling *feedback* which *reinforces* positive or required behaviour change; by providing incentives, reminders, and behaviour *prompts* for action; and by inducing negative feelings (*fear*) regarding surveillance, stigma, and punishment. Thus, visibility is not only about relations to others (anonymity, surveillance) but also refers to visibility in relation to self, via enhanced awareness and knowledge [40].

### Limitations

The approach adopted in this review has advantages over quantitative systematic reviews as qualitative studies offer access to understanding and testing the underlying mechanisms through which telehealth interventions operate. The limitations of this review are that the concluding picture only offers a broad outline of the mechanisms involved in telehealth interventions, which needs further validation and testing in different contexts and in relation to other conditions. Future work needs to also examine whether and how the relative importance of each of the three mechanisms might vary depending on, for example, type of condition, multi-morbidities, structure of personal networks, and broader social and physical environment. Such insights could contribute to building a richer and more nuanced conceptual framework of relevant mechanisms and feed into developing robust methods for telehealth development, implementation, and evaluation. Such methods could be used in addition, or as an alternative, to the currently dominant trials and quantitative techniques.

We recognise that using a wider set of concepts in our search strategy might have brought papers that merited inclusion. However, while this might have potentially added further nuance to our findings, the extensive review process that led to generating the three key concepts and the papers reviewed as a way of testing them gives us confidence in the validity of our findings.

## Conclusion

In this review, we tested processes that contribute to the successful implementation of telehealth interventions. The three mechanisms that we describe are relevant for assessing the likely implementability of existing interventions and for developing new ones that are more likely to be successfully implemented. The review draws attention to key aspects of assessing interventions likely implementability. These can be used as a basis for developing a set of sensitising concepts when consideration is being given to introducing new tele-health interventions.

Our review has three implications for developing and the future successful implementation of telehealth interventions based on the upfront consideration given to the mechanisms that are most likely to ensure whether and how the telehealth intervention enables or limits the possibility for relationships with professionals and or peers, fits with existing environment and capacities to self-manage and visibility enabling enhanced awareness to self and others which feeds into motivation and facilitates actions. If the intervention removes or replaces relationships, then other mechanisms for support and reinforcement may be necessary to effect behaviour change. Given the apparent importance of relationality, it seems likely that patients and professionals might resist or reject interventions which threaten or limit these opportunities. Second, *successful telehealth interventions are those that can be well integrated into everyday life and healthcare routines*. Interventions which enhance or improve access to care (by, for example, enabling access or making it more timely) are more likely to be acceptable to patients. Ease of use is important for adoption of technologies. Our synthesis suggests that the intervention comparatively simple technology (for example, telephone) makes for easier access and use. The intervention should be designed so that it offers minimal disruption to patients’ lives and professional routines. Third, *the design of the telehealth intervention should address the issue of visibility*. How it does this may depend on the condition and patient group involved. Monitoring systems can offer opportunities for visible feedback and prompts to actions which serve as reinforcement of behaviour change. This may be especially important if relationships are not fostered by the intervention (that is, monitoring may be used to mitigate the loss of relationship with healthcare professional). While this review identified positive aspects of visibility, the earlier reviews we conducted indicated that visibility may also have negative dimensions. Specifically, patients with mental health may wish to remain anonymous when using the system [45,47]. The design of the telehealth intervention should consider if and how symptoms and signs are made visible by the system and how these are responded to by the technology, the patient, and the healthcare professional. Further research might need to identify factors that may help maintain these technologies over the long term, which is currently underresearched.
